# Human Induced Pluripotent Stem Cell-Derived Exosomes as a New Therapeutic Strategy for Various Diseases

**DOI:** 10.3390/ijms22041769

**Published:** 2021-02-10

**Authors:** Aline Yen Ling Wang

**Affiliations:** Center for Vascularized Composite Allotransplantation, Chang Gung Memorial Hospital, 5 Fu-hsing Street, Gueishan, Taoyuan 333, Taiwan; aline2355@yahoo.com.tw

**Keywords:** induced pluripotent stem cells, exosome, cell-free regenerative medicine

## Abstract

Recently, an increasing number of studies have demonstrated that induced pluripotent stem cells (iPSCs) and iPSC-derived cells display therapeutic effects, mainly via the paracrine mechanism in addition to their transdifferentiation ability. Exosomes have emerged as an important paracrine factor for iPSCs to repair injured cells through the delivery of bioactive components. Animal reports of iPSC-derived exosomes on various disease models are increasing, such as in heart, limb, liver, skin, bone, eye and neurological disease and so forth. This review aims to summarize the therapeutic effects of iPSC-derived exosomes on various disease models and their properties, such as angiogenesis, cell proliferation and anti-apoptosis, with the hopes of improving their potential role in clinical applications and functional restoration.

## 1. Introduction

Since Yamanaka et al. discovered the delivery of Oct3/4, Sox2, c-Myc and Klf4 into somatic fibroblasts using retrovirus systems, it has become clear that the fibroblasts can be reprogrammed into pluripotent stem cells and are thus defined as induced pluripotent stem cells (iPSCs) [1]. The discovery of this reprogramming technique won him a Nobel Prize in Physiology or Medicine in 2012. iPSCs possess regenerative properties and have the ability to differentiate into any cell lineage in the body. This property is similar to that of embryonic stem cells (ESCs), although iPSC generation does not face the ethical controversies associated with sources of ESCs. Cell sources for iPSC reprogramming include somatic fibroblasts, peripheral blood mononuclear cells and even mesenchymal stem cells such as adipose-derived stem cells. These are easier to harvest than embryos and do not involve ethical controversies. Therefore, the use of iPSCs has great promise in regenerative medicine [2]. For example, the overexpression of four pluripotent factors converts fibroblasts from diseased patients into iPSCs using reprogramming techniques. The mutated genes of diseased iPSCs can be repaired using the homologous recombination method and differentiated into therapeutic somatic cells and then sequentially transferred into diseased patients for cell therapy. In addition, iPSCs generated from healthy or diseased cells can also be used for the in vitro screening of drug candidates [3,4,5,6,7,8]. In consideration of the possibility of eliciting alloimmune responses towards allogeneic iPSCs, autologous iPSC-differentiated therapeutic cells are preferred for use in diseased patients. This shows great promise for precision and personalized medicine.

In addition to utilizing the regenerative property of iPSCs for disease treatment, iPSCs also produce some modulatory factors for the direct regulation of neighboring cells. iPSCs were demonstrated to upregulate nerve growth factors such as neurotrophic factor 3, promoting sciatic nerve recovery and regeneration [9]. These modulatory factors from iPSCs can be released in the form of extracellular vesicles, such as exosomes. Thus, iPSC-secreted exosomes inspire a therapeutic strategy without cell administration for diseased patients. Pluripotent factors were also demonstrated in iPSC-derived exosomes (iPSC-Exos), which have great potential for cell-free regenerative medicine. Recently, studies have shown that iPSC-Exos exhibit therapeutic efficacy in various disease models, suggesting that iPSC-Exos have great potential as an alternative therapy for diseases. Therefore, in this review, we summarize the therapeutic applications and potential properties of iPSC-Exos in various disease models and tissues, respectively.

## 2. Exosomes

### 2.1. Biogenesis, Secretion and Delivery of Exosomes

Extracellular vesicles (EVs) play an important role in intercellular communication between cells and organs. EVs contain exosomes (diameter range: 60–180 nm), microvesicles (diameter range: 50–1000 nm) and apoptosomes (diameter range: 50–5000 nm) [10]. Exosomes are secreted by most cell types [11,12] and include a variety of proteins and nucleotides [13]. For example, injured organs secrete stimulatory signals to induce stem cells to produce healing RNAs and protein-containing exosomes, which facilitate the maintenance of tissue homeostasis. Exosome compositions are influenced by inflammatory signals such as lipopolysaccharide, tumor necrosis factor-α, interferon-γ and hypoxia. In addition, other physiological factors and cellular conditions also affect exosome release, such as intracellular levels of calcium, cellular energetics, membrane phospholipid components, membrane-acting enzymes, cytoskeleton–membrane interactions and other effectors of exocytosis, hypoxia and oxidative stress [14,15]. The exosomes are loaded with bioactive components for intercellular communication and then gradually mature as they are delivered into multivesicular bodies (MVBs) by inward budding [16]. This process prevents exosomes from degradation by cytoplasmic lysosomes. MVBs then fuse with the plasma membrane and are secreted into the microenvironment, as is the case for blood, amniotic fluid, breast milk and malignant ascites fluids [17]. Thus, exosomes contain distinct subsets of RNAs and proteins depending on the cell type from which they are secreted, making them useful for biomarker discovery.

In addition, circulating exosomes are also recognized by a variety of receptors on recipient cells which, after being taken up, receive the exosome cargo. Exosome uptake may occur through three mechanisms: endocytosis, ligand–receptor uptake and fusion. Exosome uptake mainly occurs by the endocytic pathway, which allows for fusion with the endosomal membrane or lysosomal targeting for degradation [18]. Endocytic pathways contain endocytosis and clathrin-independent pathways, such as phagocytosis, macropinocytosis, caveolin-mediated uptake and lipid raft-mediated internalization [19]. Ligand–receptor-mediated exosome internalization can specifically deliver bioactive components into target cells [20,21]. For example, ligand proteins expressed on the exosome surface, such as integrins, CD9, CD63 and CD81, are readily internalized by specific receptors on target cells and message delivery is mediated between cells through the release of exosome cargo into the cytoplasm or nucleus of the target cells [22]. Fusion-mediated exosome cargo delivery involves the fusion of the exosome with the cell membrane, releasing the cargo into the cytoplasm of the target cells [23].

### 2.2. Components of Exosomes

In general, the diameter range of exosomes is 60–180 nm and their major components are proteins, lipids and nucleic acids [24,25]. The architecture of exosome membranes is a lipid bilayer composed of high levels of sphingomyelin, cholesterol and phosphatidylserine. These unique lipid bilayer membranes of exosomes possess the ability to protect the bioactive components from degradation by cytosolic enzymes and enable the delivery of cargo into target cells [26,27]. Thus, exosomes are extracellular organelles that facilitate communication between cells and organs.

The most common proteins in exosomes include those from endosomes, the cytosol and cell membranes but do not include those from the cell nucleus, mitochondria, endoplasmic reticulum and Golgi complex. Four types of proteins make up the exosome membrane: tetraspanins, adhesion proteins, antigen presentation proteins and membrane transport and fusion protein. Tetraspanins have a function in exosome formation and secretion and include proteins such as CD9, CD63 and CD81 [28]. Adhesion proteins have a function in exosome maturation and target cell binding and include proteins such as integrins, intercellular adhesion molecule 1 (ICAM-1) and CD31. Antigen presentation proteins have a function in immune modulation, anergy and priming and include proteins such as major histocompatibility complex (MHC) classes I and II. Membrane transport and fusion proteins have functions in exosome biogenesis, secretion and downstream cell fusion, an include proteins such as SNAP, annexins and Ran5b. Thus, these four types of proteins can be used as markers for exosome characterization. In addition, enzymes and heat shock proteins are also included in exosomes. Nucleic acids such as DNA fragments, mRNA, microRNAs (miRNAs) and non-coding RNAs are found within exosomes as well [29].

## 3. Application of hiPSCs-Derived Exosomes in Diseases

### 3.1. Cardiovascular Disease

iPSC-Exos have been reported to serve as therapeutic agents, signaling mediators and pathogenic mediators in cardiovascular diseases. A previous study demonstrated that ESC-derived exosomes promote endogenous repair and enhance cardiac function following myocardial infarction (MI) [30]. In recent years, emerging studies have also shown the effective therapeutic effects of iPSC-derived exosomes in MI models. Wang et al. [31] isolated exosomes from mouse iPSCs and sequentially administered them in a mouse ischemic myocardium model with intramyocardial injection. miPSC-Exos showed cardiac protection against myocardial ischemia/reperfusion (MIR) injury. In vitro studies indicate that miPSC-Exos have anti-apoptotic and anti-oxidative effects, for example, protecting H9C2 cells against H_2_O_2_-induced oxidative stress by inhibiting caspase 3/7 activation. These cardioprotective effects were demonstrated to be associated with the delivery of protective miRNA such as Nanog-regulated miR-21 and hypoxia-inducible factor (HIF)-1α-regulated miR-210 to H9C2 cardiomyocytes in vitro. Gao et al. [32] demonstrated that exosomes from human induced pluripotent stem cell-derived cardiomyocytes (iCM) also have cardioprotective effects in a swine MI model by the measurement of left ventricular ejection fraction, wall stress, myocardial bioenergetics and cardiac hypertrophy. These exosomes exhibited anti-apoptotic and angiogenic functions. In vitro studies also showed angiogenic and anti-apoptotic effects depending on increased endothelial cell tube formation and cardiomyocyte survival derived from hiPSCs. Interestingly, exosomes from iCM improved myocardial recovery without increasing the frequency of arrhythmogenic complications, suggesting that hiPSC-Exos may provide a cellular therapeutic option for myocardial injury. Santoso et al. [33] also used exosome therapy from iCM in a mouse MI model. iCM-Exos can significantly improve MI by reducing apoptosis and fibrosis. In vitro studies demonstrated the anti-apoptotic effects by reducing the apoptosis of iCM.

In addition, EVs derived from miPSCs, hiPSC cardiomyocytes, hiPSC cardiovascular progenitors and hESC cardiovascular progenitors significantly improved cardiac repair [34,35,36]. These EVs displayed the properties of angiogenesis, anti-apoptosis and the migration and delivery of cardioprotective miRNA for tissue repair.

hESC-CM-Exos and hiPSC-CM-Exos were further investigated and showed a similar exosome profile of abundant miRNAs, including several miRs associated with cardioprotection such as miR-1, miR-21 and miR-30 [37,38,39,40]. A comparative analysis showed a decrease in miR-22 expression in exosomes from cardiac fibroblast-derived hiPSCs compared with dermal fibroblast-derived hiPSC exosomes [41]. miR-22 is reported to be elevated in cardiac hypertrophy and remodeling [42]. miR-22 overexpression can induce cardiomyocyte hypertrophy. The specific loss of miR-22 in hearts blunted cardiac hypertrophy and cardiac remodeling in response to stress. Thus, miR-22 plays a critical role in the regulation of cardiomyocyte hypertrophy and cardiac remodeling.

CD82 is reported to function in exosome production [43] and it has been further demonstrated that CD82 contributes to cardiomyocyte differentiation from hiPSCs by attenuating the Wnt/β-catenin signaling pathway through exosomal regulation [44]. Wnt signal inhibition through the exosomal clearance of β-catenin from the cells is mediated by exosome-mediated β-catenin excretion [45]. The inhibition of the Wnt signal is associated with the differentiation of cardiomyocytes from mesoderm cells [46,47].

One study reported that exosomes from the serum of pediatric dilated cardiomyopathy (DCM) patients can induce pathological changes in gene expression in neonatal rat ventricular myocytes (NRVMs) and hiPSC-CMs [48]. This suggests that DCM serum exosomes mediate pathological responses in cardiomyocytes and may contribute to the disease process of pediatric heart failure. DCM serum exosomes may be used as a potential therapeutic target specific to DCM patients.

From these results, exosomes secreted from iPSCs or iPSC-derived cardiomyocytes both exhibit cardiac protective effects by the delivery of protective molecules into injured target cells that regulate apoptosis, inflammation, fibrosis and angiogenesis.

### 3.2. Liver Disease

Several studies have shown the hepatoprotective effects of exosomes from hiPSC-derived mesenchymal stromal cells (MSCs) against hepatic ischemia–reperfusion (I/R) injury. Nong et al. [49] and Du et al. [50] demonstrated that exosomes from hiPSC MSCs display hepatoprotective effects in rat and mouse hepatic I/R injury models, respectively. The hiPSC-MSC-Exos group showed a decrease in hepatocyte necrosis, sinusoidal congestion and the hepatocyte injury markers aspartate aminotransferase (AST) and ALT (alanine aminotransferase) compared with the control group. Nong et al. concluded that hiPSC-MSC-Exos ameliorate hepatic I/R injury, possibly via the suppression of inflammatory mediators such as tumor necrosis factor alpha (TNF-α) and interleukin-6 (IL-6), the attenuation of the oxidative stress response such as glutathione (GSH), glutathione peroxidase (GSH-Px) and superoxide dismutase (SOD) and the inhibition of apoptosis such as caspase-3 and bax. Du et al. concluded that hiPSC-MSC-Exos could alleviate hepatic I/R injury by activating sphingosine kinase (SK1) and the sphingosine-1-phosphate (S1P1) pathway in hepatocytes to promote cell proliferation. Furthermore, the inhibition of the SK1 or S1P1 receptor completely abolished the protective and proliferative effects of hiPSC-MSC-Exos on hepatocytes. These results represent a strategy that could potentially promote liver regeneration and has implications for alternative therapeutic approaches to acute liver disease.

### 3.3. Limb Disease

In ischemic limb disease, Hu et al. [51] found that the intramuscular injection of exosomes from hiPSCs MSCs can attenuate mouse limb ischemia by observing enhanced microvessel density and blood perfusion, promoting angiogenesis. These exosomes could activate angiogenesis-associated molecule expression and promote human umbilical vein endothelial cell (HUVEC) migration, proliferation and tube formation. To further investigate the mechanism of exosome-mediated protection from limb ischemia, Ye et al. [52] isolated exosomes from hiPSC-derived endothelial cells (ECs) and intramuscularly injected them into a mouse ischemic hindlimb. They found that hiPSC-EC-Exos could induce HUVEC migration, proliferation and tube formation in vitro and enhance microvessel density and blood perfusion in ischemic limbs in vivo. As the underlying mechanism, it was demonstrated that hiPSC-EC-Exos contain high levels of miR-199b-5p and subsequently induce angiogenesis, with the observation of cell migration, proliferation and tube formation through the Jagged-1-dependent upregulation of vascular endothelial growth factor receptor 2 (VEGFR2) in human umbilical vein endothelial cells (HUVECs). The function of miR-199b-5p was documented as angiogenesis and as an miRNA suppressor of Jagged1 in ovarian cancer [53,54]. hiPSC-EC-Exos enriched with miR-199b-5p enhanced VEGF2 expression and promoted VEGF2-induced angiogenesis through the inhibition of Jagged 1/Notch1 signaling-mediated VEGF2 suppression via the transcriptional suppressor hairy and enhancer of split 1 (HES-1).

### 3.4. Skin Disease

Several works have shown the effective application of iPSC-Exos in skin disease, such as wound healing. Zhang et al. [55] provided the first evidence for the potential of hiPSC-MSC-Exos in treating rat cutaneous wounds. The subcutaneous injection of hiPSC-MSC-Exos around rat wound sites resulted in accelerated re-epithelialization, reduced scar widths and the promotion of collagen maturity. Exosome treatment stimulated the proliferation and migration of human dermal fibroblasts and HUVECs and increased type I and III collagen and elastin secretion in a dose-dependent manner in vitro. Their findings suggest that hiPSC-MSC-Exos can facilitate cutaneous wound healing by promoting collagen synthesis and angiogenesis. Diabetic ulcer mice treated with exosomes from undifferentiated hiPSCs also had faster wound closure and healing rates, as demonstrated by the study of Kobayashi et al. [56]. Their exosomes had the ability to promote fibroblast migration and proliferation in an in vitro scratch assay.

To investigate the potential application of exosomes derived from iPSCs in human clinical trials, Lu et al. [57] treated exosomes derived from autologous and allogeneic rhesus macaque iPSCs in wounds and found evidence of accelerated skin wound healing in both groups, as demonstrated by wound closure, epithelial coverage, collagen deposition and angiogenesis. The exosomes promoted the cell viability of injured epidermal, endothelial and fibroblastic cells in vitro.

Macaque iPSC-Exos contained low levels of pluripotent mRNAs such as Oct4, Sox2, Klf-4 and Nanog, whereas they did not deliver pluripotency to host cells. A possible explanation for this was the transient presence of pluripotent mRNAs in exosome-receiving cells and the delivered mRNAs were not sufficient to be translated into a detectable amount of pluripotent factors. Exosome-receiving cells thus did not have the reprogramming ability, suggesting that macaque iPSC-Exos carry no risk of forming teratomas. Moreover, allogeneic exosomes did not elicit lymphocyte infiltration into the skin lesion, as demonstrated by Western blot assay for the T-cell marker CD3, B cell marker CD20 and monocyte/macrophage marker CD68. This suggests that allogeneic iPSC-Exos may be the preferred choice for “off-the shelf” iPS cell-free products due to their mass production with no concern for teratoma formation. Allogeneic iPSC-derived exosomes may represent a promising alternative approach for disease therapy in addition to personalized medicine using autologous exosomes. Therefore, the potential application of allogeneic iPSC-Exos in various disease models needs more study and evidence.

### 3.5. Neurological Disease

iPSC-Exos were reported to serve as pathogenic mediators, therapeutic agents and biomarkers in neurological diseases. Alzheimer’s disease (AD) is characterized by the progressive accumulation of the aggregation-prone proteins amyloid- (A) and hyperphosphorylated-tau (p-tau) [58,59,60]. Winston et al. [61] demonstrated that exosomes derived from hiPSC neurons that express the repeat domain of tau P301L and V337M mutations (NiPSCEs) would induce pathogenesis in a wild-type mouse brain. The presence of tau inclusions throughout the brain, the increase in phosphorylated tau immunoreactivity and the extensive degeneration of neuronal dendrites in both the ipsilateral and contralateral hippocampi were observed in NiPSCEs-treated mice. This suggests that exosomes are sufficient to cause the long-distance propagation of tau pathology and neurodegeneration in vivo. To further understand the dysregulation of pathogenic exosomes, they were analyzed using proteomics and bioinformatics in the study of Podvin et al. [62]. They found that the expression of the P301L and V337M mutations of tau in human iPSC neurons results in the recruitment of distinct proteins to exosomes. mTau can be a dynamic regulator of the biogenesis of exosomes, resulting in acquisition, deletion and the upregulation or downregulation of protein cargo, causing pathogenic mTau exosomes to be capable of the in vivo propagation of p-Tau neuropathology in the mouse brain.

In addition, exosomes were demonstrated to regulate neurogenesis and neural circuit assembly in the study of Sharma et al. [63]. They examined the protein cargo and signaling bioactivity of exosomes released from hiPSC-derived neurons lacking methyl-CpG binding protein 2 (MECP2), a model of the neurodevelopmental disorder known as Rett syndrome, with exosomes released by isogenic rescue control neurons. Treating MECP2-knockdown human primary neurons with control exosomes reduces deficits in neuronal proliferation, differentiation, synaptogenesis and synchronized firing, whereas exosomes from MECP2-deficient hiPSC neurons lack this capability.

Aberrant hexanucleotide repeat expansions in C9orf72 are the most common genetic change underlying amyotrophic lateral sclerosis (ALS) and frontotemporal dementia (FTD). RNA transcripts containing these expansions undergo repeat associated non-ATG RAN translation to form five dipeptide repeat proteins (DPRs). In spinal motor neuron cells derived from induced pluripotent stem cells from C9orf72-ALS patients, Westergard et al. found the cell-to-cell spreading of DPRs through exosome-dependent and -independent pathways, which may potentially be related to disease [64].

Bipolar I disorder (BP) is a serious, recurrent mood disorder that is characterized by alternating episodes of mania and depression. To investigate novel approaches for BP patients, iPSCs were generated from the skin samples of BP patients. Attili et al. established the iPSC model of BP using exosomes derived from iPSC astrocytes [65].

The therapeutic potential of EVs from hiPSCs was investigated in mouse and porcine stroke models by Webb et al. They found that iPSC neural stem cell EVs (iNSC EVs) can improve tissue and functional recovery in a mouse thromboembolic stroke model, possibly via the modulation of the systemic immune response [66]. iNSC EVs improved cellular, tissue and functional outcomes in middle-aged rodents, whereas iMSC EVs were less effective. Moreover, iNSC EVs improved motor function in the aged rodent as indicated by beam walking, instances of foot faults and strength as evaluated by the hanging wire test. In the porcine stroke model, where clinically relevant end-points were used to assess recovery in a more translational large animal model, iNSC EVs were found to significantly improve neural tissue preservation and functional levels in cases of post-middle cerebral artery occlusion (MCAO), suggesting that iNSC EVs may be a paradigm-changing therapeutic option for stroke [67].

Candelario et al. reported that two kinds of exosomes were found from different Parkinson’s disease (PD) tissue sources [68]. One exosome was isolated from human neural progenitor (AHNP) cells from the substantia nigra of postmortem PD patients and the other was isolated from the leucine-rich repeat kinase 2 (LRRK2) gene identified in patient iPSCs. This suggests that exosomes can serve as biomarkers of idiopathic PD patients (AHNPs) and mutant LRRK2 PD patients.

### 3.6. Bone Disease

iPSC-Exos have been reported to serve as tissue engineering agents and therapeutic agents in bone diseases. Emerging evidence has shown that exosomes derived from iPSCs exhibit therapeutic effects for bone diseases such as bone defects, osteonecrosis and osteonecrosis (OA). Bone defects are generally caused by trauma, severe infection, tumor resection and skeletal abnormalities and constitute major challenges in orthopedic surgery, yet there is still no effective solution to this problem. Due to the combination of the advantages of MSCs and iPSCs and without immunogenicity for hiPSC-MSC-Exos, Qi et al. implanted exosomes into critical-size bone defects in ovariectomized rats [69]. Exosome treatment could dramatically induce bone regeneration and angiogenesis in critical-sized calvarial defects in vivo, enhance cell proliferation and alkaline phosphatase activity and upregulate the mRNA and protein expression of osteoblast-related genes in bone marrow MSCs in vitro. To further the study of the osteogenesis ability of exosomes, Zhang et al. combined hiPSC-MSC-Exos with tricalcium phosphate (β-TCP) to form scaffolds for the repair of bone defects [70]. The exosome/β-TCP scaffolds could enhance osteogenesis compared with β-TCP scaffolds only via activating the PI3K/Akt signaling pathway.

In the osteonecrosis of the femoral head (ONFH), which was caused by local ischemia, Liu et al. demonstrated that hiPSC-MSC-Exos could prevent osteonecrosis by promoting angiogenesis [71]. The administration of hiPSC-MSC-Exos could significantly prevent bone loss and increase microvessel density in the femoral head in vivo and enhance the proliferation, migration and tube-forming capacities of endothelial cells in vitro, as demonstrated by the activation of the PI3K/Akt signaling pathway in endothelial cells.

In osteoarthritis (OA), Zhu et al. demonstrated that hiPSC-MSC-Exos have a better therapeutic effect on OA in vivo and better angiogenesis and migration and proliferation of chondrocytes in vitro than synovial membrane MSC-Exos [72].

### 3.7. Eye Disease

Corneal epithelial defects can cause corneal wounds and render the eye susceptible to infection, stromal ulceration, perforation and scarring, which lead to severe vision loss [73,74]. While there has been great progress in the treatment of corneal diseases such as supportive measures in the form of lubrication, antibiotics, bandage contact lenses and the amniotic membrane [75], corneal defect healing in the setting of severe corneal disease or damage remains challenging [76]. Wang et al. [77] found that hiPSC-Exos exhibited better therapeutic effects and promoted corneal epithelium defect healing in vivo. They also exhibited better in vitro effects on the proliferation, migration, cell cycle promotion and apoptosis inhibition of human corneal epithelial cells than treatment with hMSC-Exos. Both exosomes promoted cell regeneration by upregulating cyclin A and CDK2 to drive human corneal endothelial cells (HCECs) to enter the S phase of the cell cycle from the G0/G1 phase. A cell-free therapeutic strategy for treating corneal wounds and other ocular surface diseases could involve the use of iPSC-Exos dissolved in eye drops. The therapeutic applications of iPSC-Exos and iPSC-derived cell exosomes in various disease models are summarized in Table 1.

## 4. Therapeutic Effects of hiPSC-Derived Exosomes on Cells In Vitro

### 4.1. Endothelial Cells

iPSC-Exos were reported to possess therapeutic effects and were manipulated in endothelial cells. Recently, scientists utilized exosome manipulation for further therapeutic applications, in addition to the direct administration of exosomes for various disease models. RNA interference (RNAi) can specifically silence target genes by the recognition and subsequent degradation of specific mRNA sequences. Due to some obstacles for RNAi, such as immunogenicity, instability and toxicity problems and difficulties crossing biological membranes, the therapeutic application of RNAi has been limited [78]. Ju et al. [79] isolated exosomes from urine exfoliated renal epithelial cells into hiPSCs and used hiPSC-Exos as an RNAi delivery system. The proinflammatory activation of pulmonary microvascular endothelial cells resulted in the continuous expression of cellular adhesion molecules and subsequently recruited neutrophils to form firm neutrophil–endothelium (PMN–EC) adhesions for further inflammatory immune responses. Thus, they used electroporation to transfer the RNAi of intercellular adhesion molecule-1 (ICAM-1) into hiPSC-Exos to form an Exo/siRNA complex. Interestingly, the Exo/siRNA complex efficiently delivered ICAM-1 siRNA into HMVECs, causing specific gene silencing that led to the inhibition of ICAM-1 protein expression and PMN–EC adhesion induced by lipopolysaccharide (LPS). These results suggest that hiPSC-Exos could be used as a natural gene delivery system to transfer therapeutic siRNAs for the suppression of inflammatory immune responses in target cells. Exo/siRNA complexes can also be applied in other disease models, although this requires more supportive evidence.

Due to the ALIX protein being involved in exosome biogenesis and cell degeneration, Sun et al. utilized ALIX manipulation for a dramatic enhancement of iPC-Exo production [80]. They used CRISPR-Cas9 and lentiviral transduction systems to generate respective iPSCs with knocked-out ALIX and overexpressed ALIX, which produced exosomes named exosome-KO and exosome-over, respectively. Exosome-over showed increased protein levels, while exosome-KO contained fewer protein types. However, the uptake of exosome-KO was not influenced by endothelial cells. Although exosome-over and exosome-KO had stronger and weaker effects on cells, respectively, both exosomes were protective for endothelial cell injury caused by hydrogen peroxide or cisplatin, as demonstrated by the promotion of cell viability, horizontal migration, angiogenic sprouting from aortic rings, the formation of capillary-like structures, inhibition of apoptosis and maintenance of the permeability of the endothelial monolayer. Interestingly, exosome-over can more effectively promote cell viability than exosome-GFP in a dose-dependent manner. These results demonstrated that ALIX overexpression can increase the therapeutic function of iPSC-Exos and the manipulation of iPSCs with ALIX overexpression can produce exosomes with a more beneficial protein content.

In addition, Ding et al. showed that hiPSC-Exos have a protective effect on high glucose-induced injury to human endothelial cells, as demonstrated by restored cell viability and capillary-like structure formation and reduced senescence in HUVECs [81]. Dougherty et al. found that hiPSC cardiomyocyte Exos possessed angiogenic properties via the promotion of tube formation, wound closure and cell proliferation in bovine aortic endothelial cells [82].

### 4.2. Fibroblasts

iPSC-Exos have been reported to possess therapeutic effects and can be manipulated in fibroblasts. Skin aging is mainly caused by intrinsic and extrinsic aging. Intrinsic aging is genetically decided; it occurs inevitably as time passes. The literature has shown that epigenetic changes and post-translational mechanisms are more important pathways of intrinsic aging than genetic influences [83]. However, extrinsic aging occurs due to external factors such as smoking, air pollution and unbalanced nutrition. Of these, UV exposure is the most important cause of extrinsic aging [84,85,86]. Therefore, several studies used exosomes derived from hiPSCs to treat aged skin fibroblasts for the prevention of skin aging. Oh et al. demonstrated that hiPSC-Exos treatment can suppress the damage of aged human dermal fibroblasts (HDFs) and inhibit matrix-degrading enzymes (MMP-1/3) caused by UVB irradiation [87]. hiPSC-Exos also reduced the expression level of SA-β-Gal and restored collagen type I expression in senescent HDFs. Kim et al. used the exosomes derived from hiPSC-MSCs to further investigate their therapeutic potential on skin aging and the underlying mechanisms [88]. They found that hiPSC-MSC-Exos could increase human keratinocytes (HaCaT) proliferation, enhance the collagen secretion of HaCaT and induce the upregulation of fibronectin in HaCaT compared to human Wharton’s jelly MSC-Exos. hiPSC-MSC-Exos can enhance the phosphorylation of extracellular signal-regulated kinase (ERK)-1/2.

Although hiPSC-Exos treatment can ameliorate skin aging, the low production yield of exosomes still limits their clinical application. To overcome this issue, Lee et al. generated cell-engineered nanovesicles (CENVs) by the serial extrusion of hiPSCs through membrane filters with diminishing pore sizes [89]. The hiPSC CENVs exhibited similar characteristics to the hiPSC-Exos but the production yield was dramatically increased. hiPSC CENVs promoted the proliferation and migration of senescent HDFs and restored senescence-associated alterations of gene expression. hiPSC CENVs significantly inhibited the expression of p53 and p21, which are key factors involved in cell cycle arrest, apoptosis and cellular senescence signaling pathways. hiPSC CENVs may provide an alternative approach to hiPSC-Exos.

### 4.3. Cardiomyocytes

Duchenne muscular dystrophy (DMD) is caused by a dystrophin deficiency (Dys), which results from mutations in the DMD gene. DMD is characterized by severe limb and diaphragm muscle weakness and patients lose independent ambulation and develop respiratory weakness within the first and second decades of their life, respectively [90].

In the second to third decade of life for these patients, cardiomyopathy will occur, subsequently representing a leading cause of death [91]. Dys hiPSC-cardiomyocyte-Exos exhibited cardioprotection via the activation of ERK1/2 and p38 MAPK signaling, as demonstrated by decreased reactive oxygen species and delayed mitochondrial permeability to maintain the mitochondrial membrane potential and inhibit cell death [92]. Interestingly, the acute effects of exosomes on target cells can be initiated from exosome surface proteins and not necessarily their internal cargo. The therapeutic effects of hiPSC-derived exosomes on cells in vitro are summarized in Table 2.

## 5. Possible Challenges for Exosomes

Exosomes provide a feasible alternative cell-free therapy in iPSC medicine. However, iPSC-Exos face some possible challenges such as their production, stability, half-life and delivery efficiency. The low yield of exosomes limits their wide use. Exosome manipulation has been demonstrated to increase exosome production by the overexpression of the ALIX protein [80]. Exosome secretion can also be enhanced by treatment with Ca^2+^ ionophores [24,93]. In cortical neurons, exosomes can be stimulated by neurotransmitters. Rab GTPases such as RAB11, RAB35, RAB27A and RAB27B are involved in exosome production through vesicle budding [94]. The expression of exosome markers such as CD63, CD81 and MHC-II was shown to be decreased by the silencing of RAB27A and RAB27B. Exosome compositions are strongly influenced by inflammatory signals such as LPS, tumor necrosis factor (TNF)-α, interferon (IFN)-γ and hypoxia.

The stability of exosomes is associated with their zeta potential, which is a physical property of exosomes obtained by measuring the magnitude of the electrostatic charge repulsion or attraction between particles [31]. Stable nanoparticles are those with absolute values of zeta potential in the range of 31 to 40 mV [95]. iPSC-Exos have been reported to have a zeta potential of −14 mV to −15 mV at 23 °C, showing a tendency for aggregation^31^. Sonication can be used to disperse aggregated exosomes [96,97]. Temperature can influence the zeta potential, which increases by around 0.39% per °C [98], suggesting that iPSC-Exos might exhibit increased stability in vivo.

Exosomes include various proteins and RNA and have short half-lives. Therefore, the optimal storage of exosomes is important. Plasma can be frozen before exosome purification and the isolated exosomes can be frozen to ensure the stability of the protein and RNA. Storage temperature is also critical for exosome cargo stability and is required to maintain exosomal marker expression in the range of −20 °C to −80 °C [99,100].

Exosomes play critical roles in cell–cell communication through endocytosis, phagocytosis and membrane fusion. Exosome uptake was found to correlate with intracellular and microenvironmental acidity [101,102], suggesting that the microenvironment influences the delivery efficiency of exosomes. In the case of factors operating at the intracellular level, delivery into the correct cellular compartments while maintaining the stability, integrity and biological potency of these factors remains challenging and expensive.

## 6. Conclusions

Recently, an increasing number of studies have shown that iPSCs can exhibit their therapeutic effects mainly through a paracrine mechanism rather than differentiation into therapeutic cells and exosomes have emerged as an important paracrine factor for iPSCs to repair injured cells. Exosome therapy has been seen as a promising cell-free approach for various diseases such as heart, limb, liver, skin, bone, eye and neurological diseases and so forth. iPSCs and iPSC derivatives may serve as a source of therapeutic exosomes that can be utilized for future clinical translation to treat various diseases. This provides a platform for the safe, effective and reliable delivery of bioactive molecules to target cells. However, further analysis of the bioactive molecules inside exosomes should be further investigated in future applications.

## Figures and Tables

**Table 1:** The therapeutic applications of induced pluripotent stem cell-derived exosomes (iPSC-Exos) and iPSC-derived cell exosomes in various disease models.

Diseases	Cell Sources	Characterization	Models	Therapeutic Effects	Mechanisms	References
Heart	miPSCs	NTA (100 nm); WB (CD63, Tsg101)	Mouse myocardial ischemia/reperfusion (MIR)	Prevent cardiomyocyte apoptosis in ischemic myocardium	Cardioprotective miRNAs (Nanog-regulated miR-21 and HIF-1α-regulated miR-210)	[31]
	hiPSC cardiomyocytes, hiPSC endothelial cells, hiPSC smooth muscle cells	TEM, NTA (98 nm), WB (CD81, CD63, flotillin-1, TSTG101)	Swine myocardial infarction (MI)	Improve recovery from myocardial infarction in swine	-	[32]
	hiPSC cardiomyocytes	TEM, NTA (142 nm), WB (CD9, CD63)	Mouse myocardial infarction (MI)	Promote autophagy for myocardial repair	miRNA	[33]
Liver	hiMSCs	TEM (50–60 nm), WB (CD9, CD63, CD81)	Rat hepatic ischemia/reperfusion (I/R) injury	Display hepatoprotective effects against hepatic I/R injury	1. Anti-inflammation (TNF-α, IL-6, HMGB1) 2. Anti-apoptosis (caspase-3, bax) 3. Anti-oxidation (GSH, GSH-Px, SOD)	[49]
	hiMSCs	TEM, NTA (135 nm), WB (Alix, CD63, CD81)	Mouse hepatic Ischemia/reperfusion (I/R) injury	Protect liver against hepatic I/R injury	Activate sphingosine kinase and sphingosine-1-phosphate signaling pathway	[50]
Limb	hiMSCs	TEM (57 nm), WB (CD9, 63, 81)	Mouse limb ischemia	Attenuate limb ischemia by promoting angiogenesis	-	[51]
	hiPSC endothelial cells	TEM, NTA (95 nm), WB (CD9, CD63, TSG101)	Mouse limb ischemia	Promote postnatal angiogenesis in mouse limb ischemia	miR-199b-5p	[52]
Skin	hiMSCs	TEM (30–100 nm); WB (CD9, CD63, CD81)	Rat wound healing	Accelerate reepithelialization, reduce scar widths and promote collagen maturity	-	[55]
	hiPSCs	TEM (120 nm), Flow (CD9, CD63, CD81)	Mouse wound healing	Promote skin wound healing in diabetic ulcer mice	-	[56]
	Rhesus macaque iPSCs (autologous vs. allogeneic)	TEM, NTA (100 nm)	Rhesus macaque wound healing	Promote wound healing by autologous iPSCs and exosomes vs. their allogeneic counterparts	-	[57]
Neuron	hiPSC neurons	NTA (100 nm)	Mouse Alzheimer’s disease (AD)	Neuronal exosome-derived human Tau is toxic to target mouse neurons in vivo	-	[61]
	hiPSC neurons	TEM, NTA (55 nm), WB (Alix, Flotillin)	Mouse dentate gyrus	Regulate neurogenesis and circuit assembly	-	[63]
	hiPSC neurons	TEM, NTA (150 nm), WB (CD63), proteomics (CD81)	Proteomics and bioinformatics	Dysregulation of exosome cargo by mutant Tau expressed in hiPSC neurons revealed by proteomics	-	[62]
Bone	hiMSCs	TEM, NTA (68.7 nm), WB (CD9, CD63, CD81)	Engineered rat tissue	Exosome/tricalcium phosphate combination scaffolds can enhance bone regeneration	Activate PI3K/Akt signaling pathway	[70]
	hiMSCs	NTA (83.3 nm), WB (CD9, CD63, CD81)	Rat bone defects	Repair critical-sized bone defects through enhanced angiogenesis and osteogenesis	-	[69]
	hiMSCs	TEM, NTA (100 nm), WB (CD9, CD63, CD81)	Rat steroid-induced osteonecrosis of the femoral head (ONFH)	Prevent osteonecrosis of the femoral head by promoting angiogenesis	Activate PI3K/Akt signaling pathway	[71]
	hiMSCs	TEM, NTA (110 nm), WB (CD9, CD63, TSG101)	Mouse collagenase-induced osteoarthritis (OA)	hiMSC-Exos prevent osteoarthritis better than synovial membrane MSC-Exos	-	[72]
Eye	hiPSCs or hMSCs	TEM, NTA (100 nm), WB (CD9, CD63)	Rat corneal epithelial defect	hiPSC-Exos promote better healing of corneal epithelial defects than hMSC-Exos	Promote cell regeneration by upregulating cyclin A and CDK2 to drive HCECs to enter the S phase of the cell cycle from the G0/G1 phase	[77]

miPSCs: mouse induced pluripotent stem cells; hiPSCs: human induced pluripotent stem cells; hiMSCs: human induced pluripotent stem cell-derived mesenchymal stromal cells; TEM: transmission electron microscopy; NTA: nanoparticle tracking analysis; WB: Western blot. TEM and NTA values show the average or range of diameters of exosomes.

**Table 2:** Therapeutic effects of induced pluripotent stem cell -derived exosomes on cells in vitro.

Diseases	Cell Sources	Characterization	Models	Therapeutic Effects	Mechanisms	References
Endothelial cells	hiMSCs	TEM (122 nm), WB (CD63, TSG101, Alix)	In vitro endothelial cells	iPSC-Exos delivering siRNA attenuate intracellular adhesion molecule-1 expression and neutrophil adhesion	RNAi	[79]
	hiPSCs	-	In vitro endothelial cells	ALIX increases protein content and the protective function of iPSC-Exos	-	[80]
	hiPSCs	TEM, NTA (103 nm), WB (CD63, Alix)	In vitro endothelial cells	Protect against high glucose induced injury	-	[81]
	hiPSC cardiomyocytes	TEM, NTA (163 nm), WB (CD63, HSP70)	In vitro endothelial cells	Enhance angiogenesis in endothelial cells	-	[82]
Fibroblasts	hiPSCs	TEM, NTA (85.8 nm), Dynamic light scattering (DLS) −15.6 mV	In vitro aged human dermal fibroblasts (HDFs)	Ameliorate the aging of skin fibroblasts	-	[87]
	hiMSCs	TEM, NTA (MSCs: 167 nm, iMSCs: 147 nm), WB (CD9, CD63)	In vitro human keratinocytes (HaCaT) and human dermal fibroblasts (HDFs)	Accelerate skin cell proliferation	-	[88]
	Cell-engineered nanovesicles (CENVs) from hiPSCs	NTA (150 nm)	In vitro human dermal fibroblasts (HDFs)	Protect the senescence of dermal fibroblasts	-	[89]
Cardiomyocytes	Human dystrophin-defcient (Dys) iPSC cardiomyocytes	TEM, NTA (WT: 148 nm, Dys: 187 nm), Flow (CD63, CD81)	In vitro Dys-iCMs	Dys iCM-Exos protect Dys iCM from stress-induced injury by decreasing reactive oxygen species and delaying mitochondrial permeability to maintain the mitochondrial membrane potential and decrease cell death	Activate ERK1/2 and p38 MAPK signaling	[92]

miPSCs: mouse induced pluripotent stem cells; hiPSCs: human induced pluripotent stem cells; hiMSCs: human induced pluripotent stem cell-derived mesenchymal stromal cells; iCMs: induced pluripotent stem cell-derived cardiomyocytes; TEM: transmission electron microscopy; NTA: nanoparticle tracking analysis; WB: Western blot; WT: wild-type; RNAi: RNA interference; siRNA: small interfering RNA. TEM and NTA values show the average or range of diameters of exosomes.

## Data Availability

Not applicable.
